# Implementation determinants of safer smoking supplies in U.S. syringe services programs

**DOI:** 10.1186/s43058-025-00714-z

**Published:** 2025-03-25

**Authors:** William H. Eger, Angel K. Gomez, Kirstin Kielhold, Tyler S. Bartholomew, Angela R. Bazzi

**Affiliations:** 1 School of Social Work, San Diego State University, San Diego, CA USA; 2 School of Medicine, University of California San Diego, La Jolla, CA USA; 3 AIDS United, Washington, D.C USA; 4 School of Public Health, San Diego State University, San Diego, CA USA; 5 Herbert Wertheim School of Public Health, University of California San Diego, La Jolla, CA USA; 6 Department of Medicine, Miller School of Medicine, University of Miami, Miami, FL USA; 7 School of Public Health, Boston University, Boston, MA USA

**Keywords:** Safer smoking supplies, Smoking, Syringe services programs, Implementation, Opioids

## Abstract

**Background:**

The prevalence of smoking opioids and other unregulated drugs has increased across the United States (U.S.) since 2000. Improved access to safer smoking supplies may reduce the health consequences of inhalation while helping to engage more people who use drugs in syringe services programs (SSPs); however, the landscape of safer smoking supply implementation is understudied.

**Methods:**

From November 2023–January 2024, we surveyed representatives of U.S. SSPs to assess safer smoking supply implementation across contextual domains of the Exploration, Preparation, Implementation, and Sustainment (EPIS) framework. Descriptive statistics were used to describe determinants across the phases of safer smoking supply implementation. Poisson regression identified factors associated with implementation.

**Results:**

Among 118 organizations responding to the survey, most received state funding (83%), were community-based organizations (CBOs; 74%), and served urban jurisdictions (62%). The majority (67%) were already providing safer smoking supplies; 16% were exploring implementation and 11% were not. On average, safer smoking supply implementation occurred more recently than the provision of syringes (1–2 years ago vs. > 5 years ago), with participant request being the most common motivation for implementation (84%). Additional facilitators of safer smoking supply implementation were organizational prioritization (65%) and internal leadership support (57%). Factors significantly associated with safer smoking supply implementation included being from the Northeastern or Western regions (vs. the U.S. South), serving exurban communities, being a CBO, receiving foundation funding, receiving private donations from fundraising, and offering syringes and other injection alternatives (e.g., safer snorting supplies). Receiving federal funding, fear of external community opposition, internal leadership opposition, and respondent uncertainty about changing demand for safer smoking supplies (vs. perceiving that demand has not changed) were negatively associated with implementation.

**Conclusions:**

Determinants in the inner context, like organizational prioritization of safer smoking supplies and internal leadership support, may facilitate safer smoking supply implementation, while specific outer context factors (e.g., funding, regional policies) may inhibit implementation. Flexible policies and funding structures and further research to build and disseminate evidence on the benefits of safer smoking supplies are needed to expand the implementation and scale-up of this prevention service within U.S. SSPs.

Contributions to the literature

- Provides the first comprehensive assessment of safer smoking supply implementation across U.S. syringe services programs using the EPIS framework
- Identified key inner-context facilitators that enhance safer smoking supply implementation efforts, such as organizational prioritization and leadership support
- Highlights significant outer-context barriers to safer smoking supply implementation, including restrictive funding, community opposition, and state/federal policies
- Informs future implementation strategy development and testing for scaling up safer smoking supplies to improve the health of people who use drugs

## Background

North America is experiencing an unprecedented polysubstance use crisis driven by unregulated drugs, including illicitly manufactured fentanyl and stimulants, leading to rising overdose mortality, HIV and hepatitis C virus transmission, and other serious public health consequences [1–4]. In this context, there has been a significant change in the route of administration away from injecting unregulated drugs towards smoking them; this trend has been identified along the West Coast of North America and potentially other U.S. regions [5–9]. While this shift in drug consumption behaviors may be protective against certain adverse health outcomes [10, 11], particularly infectious disease transmission via contaminated injection preparation equipment, it also suggests a need for programmatic adaptations to support individuals in smoking drugs safely, carrying important implications for prevention programs that have traditionally focused on reducing the health harms of injection drug use.

Substantial evidence supports the effectiveness of syringe services programs (SSPs) in reducing numerous adverse health consequences of unregulated drug use [12–14]. Historically, SSPs have provided sterile syringes and other injection preparation equipment, naloxone, condoms, infectious disease testing, and supported referrals to a range of health and social services [15, 16]. SSPs thus represent a critical public health service delivery setting to address the evolving needs of people who use drugs (PWUD). More recently, many SSPs have likely adapted their services and delivery models to better address the increasing trend of smoking drug use [17]. These adaptations include the implementation of safer smoking supplies (e.g., sterile glass pipes, silicone mouthpieces, lip balm) that can help reduce injecting and increase engagement of PWUD in other types of SSP services [18, 19], potentially enhancing the reach and effectiveness of SSPs.

Despite their critical importance for public health, SSPs as a service setting remain relatively understudied in the health services and implementation science literature [20–24]. In addition, there are significant research gaps related to the overall state of safer smoking supply implementation through U.S. SSPs [17], including a dearth of knowledge on the factors that drive—and inhibit—organizations’ abilities to implement this type of intervention. Furthermore, little is known about SSPs’ decision-making, motivations and implementation processes relating to safer smoking supplies, including the inner- and outer-contextual determinants of implementation that could inform future strategies to support implementation and sustainment. To fill these gaps, we drew from the Exploration, Preparation, Implementation, and Sustainment (EPIS) framework [25, 26] to investigate the current landscape of safer smoking supply implementation within SSPs across the U.S.

## Methods

### Study design, sample, and data collection

In summer 2023, collaborators at AIDS United (a national nonprofit organization focused on ending the HIV epidemic and improving the lives of people with and at risk for HIV) initiated this project in response to concerns raised by community members and funding recipients regarding the procurement of safer smoking supplies. After in-depth discussions regarding recruitment and survey development, we partnered with AIDS United to recruit representatives of U.S. SSPs and other harm reduction organizations (i.e., organizations providing prevention services to PWUD) who were at least 18 years of age and could read English to participate in a one-time, anonymous survey. Recruitment occurred from November 2023 to January 2024 by sending emails within our professional networks, through AIDS United’s list of funded SSPs and known collaborators, and by requesting that enrolled respondents refer their professional contacts at other organizations via snowball sampling. Recruitment emails briefly explained the study, requested that only one representative participate per organization, and provided a link to electronic screening and consent procedures. After consenting to participate, respondents were directed to an anonymous survey in Qualtrics (Provo, UT). Respondents did not receive compensation for completing the survey, which took a median of 13 min. The UC San Diego Institutional Review Board (IRB) reviewed and approved all study activities and provided a Not Human Subjects Research determination.

### Survey measures

Guided by the EPIS framework [25, 26], our primary dependent variable was SSPs’ current safer smoking supply implementation phase (i.e., step in the process of implementing safer smoking supplies) with categorical responses for: not currently exploring safer smoking supply implementation, currently exploring safer smoking supply implementation but not yet offering any smoking supplies, preparing for safer smoking supply implementation in the next six months, and currently implementing (i.e., offering) safer smoking supplies [25]. Also drawing on EPIS, other key survey domains included: (1) organizational characteristics, (2) safer smoking supply implementation determinants, (3) additional safer smoking supply measures, and (4) sustainment and scale-up considerations. Respondents could decline to answer any question.

#### Organizational characteristics

Included the organization’s state (or territory) of operation, which included all 50 U.S. states, Washington D.C., and Puerto Rico (recategorized to Midwest, Northeast, West and South); urbanicity of areas served (urban, suburban, exurban/semi-rural, rural); type of organization (community-based organization [CBO]/non-profit, health department (city, county, or state), or other); funding sources (federal, state, foundation, private donations from fundraising, or other); duration of operation (in years); the number of unique clients served (past month); services directly offered by the organization; and for organizations providing syringes, duration of time providing them (in years).

#### Safer smoking supply implementation determinants

Asked of all respondents included respondents’ perceptions regarding changing community demand for safer smoking supplies in the past six months (increased, decreased, unsure, remained the same) and the following outer and inner contextual determinants: the *outer context* included local policies (e.g., paraphernalia laws), federal policies (e.g., inability to purchase safer smoking supplies with federal dollars), opposition external to the organization (from local organizations, leaders external to the organization, or the community), fear of external pushback (from local organizations, leaders external to the organization, or the community), and insufficient funding. The *inner context* included uncertainty regarding the evidence for safer smoking supplies, internal leadership opposition, limited staff/leadership awareness of the need for safer smoking supplies, limited interest from SSP clients, and uncertainty regarding best practices for safer smoking supply implementation from staff/leadership.

For organizations that have already implemented safer smoking supplies, we assessed motivations for implementation, including request/feedback from SSP clients, request/suggestion from a local harm reduction partner organization, guidance/technical assistance, funding opportunity, or another specific motivation (with a fill-in option). We also assessed facilitators of safer smoking supply implementation, including factors in the *outer-* (new funding specifically for safer smoking supplies, new funding not specific for safer smoking supplies, external support (e.g., from local health department), collaborator/partner support) and *inner-context* (internal leadership support, and making safer smoking supplies an organizational priority).

#### Additional safer smoking supply measures

Included the duration of time providing safer smoking supplies (in years) and which smoking supplies respondents’ organizations currently provided. For organizations currently providing any type of pipe (including bubble pipe/ “oil burners”, hammer pipes, or straight pipes), we also asked approximately how many pipes they distributed (past month), whether they set a limit on the number of pipes distributed within a single encounter (and if so, the quantity of pipes per encounter), and if SSP clients are asked or required to return used pipes to obtain new ones (i.e., “exchange” pipes).

#### Sustainment and scale-up considerations

Based on the Program Sustainability Assessment Tool [27] and the Program Sustainability Index [28], included survey respondents’ perceptions (on a 4-point Likert scale ranging from strongly disagree to strongly agree) of whether (1) their organization distributes enough safer smoking supplies to meet community needs; (2) offering safer smoking supplies is consistent with their organization’s mission; (3) their organization has a combination of stable and flexible funding for safer smoking supplies; (4) their organization’s funding is sufficient for their current safer smoking supply operations; (5) diverse external community organizations are invested in the success of their safer smoking supply program; (6) safer smoking supplies are well integrated into their organization’s operations; and (7) their organization has the capacity for quality program evaluation [29].

### Data analysis

We limited all analyses to SSPs that responded to our question about their phase of safer smoking supply implementation. We first calculated descriptive statistics to summarize organizations’ characteristics overall and by their safer smoking supply implementation status. We then dichotomized organizations’ implementation status to indicate whether a) they have or b) have not yet implemented safer smoking supplies (at the time of study participation) due to data sparseness in certain implementation phases and used chi-square and Fisher’s exact tests (for variables with cell counts ≤ 5) for categorical variables and Students’ t-tests for continuous variables to examine differences between groups. Next, to explore factors associated with safer smoking supply implementation, we fit separate modified Poisson regression models for each exposure to obtain unadjusted prevalence ratios (PRs) and corresponding 95% confidence intervals (CIs) to determine the magnitude of effect and significance [30]. Characteristics with p-values less than or equal to 0.10 in descriptive analyses were chosen for bivariate regression analyses.

Finally, for organizations currently implementing safer smoking supplies, we used proportions to descriptively examine the characteristics of these organizations and factors that respondents indicated impact the implementation and sustainment of safer smoking supplies. We used R version 4.4.0 for all statistical analyses [31].

## Results

### Characteristics of the total sample

Of 122 U.S. SSPs that responded to our survey, 118 (97%) answered our question about their safer smoking supply implementation phase. Most organizations (67%) had implemented safer smoking supplies (“implementation”), 3% were preparing to implement in the next 6 months (“preparation”), 16% were exploring implementation (“exploration”), and 11% were not exploring implementation (Table 1). In our final analytic sample, the largest proportion were in the U.S. West (41%). Most served urban jurisdictions (62%), were community-based organizations (CBOs; 74%) and received state funding (83%). The majority of organizations (72%) had been operating for > 5 years. The median number of unique monthly participants was 300 (interquartile range [IQR]: 499). Organizations offered a wide range of prevention supplies, including naloxone (99%), safer sex supplies (97%), syringes (92%), basic first aid (92%), referrals (90%), HIV (71%) and hepatitis C testing (65%), food (59%), medications for opioid use disorder coordination (57%), safer snorting (54%) and anal administration supplies (53%), and case management/housing coordination services (50%). A majority of survey respondents believed that the demand for safer smoking supplies had increased in the past six months (66%), but many also reported various barriers to safer smoking supply implementation, including insufficient funding (58%) and local (41%) and federal policy restrictions (31%). A detailed examination of each implementation phase is outlined in Table 1.

**Table 1:** Implementation phases for safer smoking supplies at SSPs in the United States, November–December 2023 (*n* = 118)

	**Pre-implementation phase not specified (*n* = 3; 2.5%)**	**Not exploring implementation (*n* = 13; 11.0%)**	**Exploring implementation (*n* = 19; 16.1%)**	**Preparing to Implement (*n* = 4; 3.4%)**	**Currently implementing (*n* = 79; 66.9%)**	**Total (*N* = 118)**^**a**^
**Organizational Characteristics**						
**Region**						
Midwest^b^	0 (0%)	2 (15.4%)	3 (15.8%)	2 (50.0%)	12 (15.2%)	19 (16.1%)
Northeast^c^	0 (0%)	1 (7.7%)	2 (10.5%)	0 (0%)	18 (22.8%)	21 (17.8%)
West^d^	1 (33.3%)	3 (23.1%)	7 (36.8%)	1 (25.0%)	36 (45.6%)	48 (40.7%)
South^e^	1 (33.3%)	6 (46.2%)	7 (36.8%)	1 (25.0%)	13 (16.5%)	28 (23.7%)
Missing	1 (33.3%)	1 (7.7%)	0 (0%)	0 (0%)	0 (0%)	2 (1.7%)
**Urbanicity of areas served**^**$**^						
Urban^&^	1 (33.3%)	8 (61.5%)	9 (47.4%)	4 (100%)	51 (64.6%)	73 (61.9%)
Suburban^&^	0 (0%)	3 (23.1%)	6 (31.6%)	1 (25.0%)	27 (34.2%)	37 (31.4%)
Exurban/Semi-rural^&^	1 (33.3%)	1 (7.7%)	3 (15.8%)	0 (0%)	25 (31.6%)	30 (25.4%)
Rural^&^	1 (33.3%)	3 (23.1%)	8 (42.1%)	1 (25.0%)	33 (41.8%)	46 (39.0%)
**Type of Organization**^**$**^						
Community-based Organization/Non-profit^&^	2 (66.7%)	6 (46.2%)	12 (63.2%)	2 (50.0%)	65 (82.3%)	87 (73.7%)
City, County, or State Health Department^&^	0 (0%)	7 (53.8%)	7 (36.8%)	1 (25.0%)	16 (20.3%)	31 (26.3%)
Other^f,&^	1 (33.3%)	0 (0%)	2 (10.5%)	0 (0%)	3 (3.8%)	6 (5.1%)
**Funding sources**^**$**^						
Federal^&^	1 (33.3%)	5 (38.5%)	15 (78.9%)	3 (75.0%)	31 (39.2%)	55 (46.6%)
State^&^	2 (66.7%)	11 (84.6%)	15 (78.9%)	4 (100%)	66 (83.5%)	98 (83.1%)
Foundation^&^	1 (33.3%)	4 (30.8%)	5 (26.3%)	1 (25.0%)	42 (53.2%)	53 (44.9%)
Private donations from fundraising^&^	1 (33.3%)	1 (7.7%)	9 (47.4%)	2 (50.0%)	43 (54.4%)	56 (47.5%)
Other^g^^,&^	2 (66.7%)	3 (23.1%)	3 (15.8%)	3 (75.0%)	22 (27.8%)	33 (28.0%)
**Duration of operation**						
Less than one year	0 (0%)	0 (0%)	0 (0%)	0 (0%)	2 (2.5%)	2 (1.7%)
1–2 years	1 (33.3%)	0 (0%)	0 (0%)	0 (0%)	5 (6.3%)	6 (5.1%)
3–5 years	0 (0%)	3 (23.1%)	4 (21.1%)	1 (25.0%)	16 (20.3%)	24 (20.3%)
Greater than 5 years	2 (66.7%)	10 (76.9%)	15 (78.9%)	3 (75.0%)	55 (69.6%)	85 (72.0%)
**Number of unique clients (past month)**						
Median [IQR]	800 [700]	300 [236]	75 [345]	150 [135]	300 [551]	300 [499]
Unsure or missing	1 (33.3%)	4 (30.8%)	10 (52.6%)	1 (25.0%)	20 (25.3%)	36 (30.5%)
**Services offered**^**$**^						
Naloxone^&^	2 (66.7%)	13 (100%)	19 (100%)	4 (100%)	79 (100%)	117 (99.2%)
Syringes^&^	2 (66.7%)	10 (76.9%)	16 (84.2%)	4 (100%)	77 (97.5%)	109 (92.4%)
Safer snorting supplies^&^	0 (0%)	1 (7.7%)	2 (10.5%)	1 (25.0%)	60 (75.9%)	64 (54.2%)
Anal administration supplies^&^	1 (33.3%)	3 (23.1%)	5 (26.3%)	1 (25.0%)	52 (65.8%)	62 (52.5%)
Basic first aid^&^	2 (66.7%)	11 (84.6%)	18 (94.7%)	4 (100%)	74 (93.7%)	109 (92.4%)
Food^&^	1 (33.3%)	6 (46.2%)	9 (47.4%)	2 (50.0%)	52 (65.8%)	70 (59.3%)
Safer sex supplies^&^	2 (66.7%)	12 (92.3%)	19 (100%)	4 (100%)	77 (97.5%)	114 (96.6%)
HIV testing^&^	1 (33.3%)	11 (84.6%)	13 (68.4%)	3 (75.0%)	56 (70.9%)	84 (71.2%)
Hepatitis C testing^&^	1 (33.3%)	10 (76.9%)	12 (63.2%)	3 (75.0%)	51 (64.6%)	77 (65.3%)
PrEP or PEP for HIV^&^	0 (0%)	4 (30.8%)	9 (47.4%)	1 (25.0%)	27 (34.2%)	41 (34.7%)
HIV treatment^&^	0 (0%)	1 (7.7%)	6 (31.6%)	0 (0%)	17 (21.5%)	24 (20.3%)
Hepatitis C treatment^&^	0 (0%)	2 (15.4%)	6 (31.6%)	0 (0%)	19 (24.1%)	27 (22.9%)
STI testing^&^	1 (33.3%)	6 (46.2%)	8 (42.1%)	2 (50.0%)	41 (51.9%)	58 (49.2%)
STI treatment^&^	0 (0%)	5 (38.5%)	8 (42.1%)	2 (50.0%)	23 (29.1%)	38 (32.2%)
Basic clinical services^&^	0 (0%)	7 (53.8%)	7 (36.8%)	1 (25.0%)	27 (34.2%)	42 (35.6%)
Mental health services^&^	0 (0%)	3 (23.1%)	11 (57.9%)	1 (25.0%)	20 (25.3%)	35 (29.7%)
Referrals^&^	2 (66.7%)	3 (23.1%)	18 (94.7%)	4 (100%)	71 (89.9%)	106 (89.8%)
Case management/housing coordination services^&^	0 (0%)	6 (46.2%)	12 (63.2%)	0 (0%)	41 (51.9%)	59 (50.0%)
MOUD coordination^&^	0 (0%)	9 (69.2%)	13 (68.4%)	1 (25.0%)	44 (55.7%)	67 (56.8%)
**Duration of providing syringes**^**h**^						
Less than one year	0 (0%)	0 (0%)	2 (10.5%)	0 (0%)	3 (3.8%)	5 (4.2%)
1–2 years	1 (33.3%)	1 (7.7%)	0 (0%)	1 (25.0%)	8 (10.1%)	11 (9.3%)
3–5 years	0 (0%)	3 (23.1%)	5 (26.3%)	1 (25.0%)	18 (22.8%)	27 (22.9%)
Greater than 5 years	1 (33.3%)	6 (46.2%)	9 (47.4%)	2 (50.0%)	44 (55.7%)	62 (52.5%)
Unsure or missing	1 (33.3%)	3 (23.1%)	3 (15.8%)	0 (0.0%)	6 (7.6%)	13 (11.0%)
**Implementation Determinants**						
**Perceived change in demand for smoking supplies (past 6 months)**						
Increased	2 (66.7%)	6 (46.2%)	10 (52.6%)	1 (25.0%)	59 (74.7%)	78 (66.1%)
Decreased	0 (0%)	0 (0%)	0 (0%)	0 (0%)	0 (0%)	0 (0.00%)
Unsure	1 (33.3%)	7 (53.8%)	4 (21.1%)	0 (0%)	1 (1.3%)	13 (11.0%)
Remained the same	0 (0%)	0 (0%)	5 (26.3%)	3 (75.0%)	15 (19.0%)	23 (19.5%)
**Barriers to implementation**^**$**^						
*Outer context*						
Local policies^&^	1 (33.3%)	5 (38.5%)	12 (63.2%)	3 (75.0%)	27 (34.2%)	48 (40.7%)
Federal policies^&^	1 (33.3%)	2 (15.4%)	7 (36.8%)	3 (75.0%)	24 (30.4%)	37 (31.4%)
External opposition^&^	2 (66.7%)	4 (30.8%)	8 (42.1%)	1 (25.0%)	14 (17.7%)	29 (24.6%)
Fear of external pushback^&^	2 (66.7%)	6 (46.2%)	7 (36.8%)	2 (50.0%)	18 (22.8%)	35 (29.7%)
Insufficient funding^&^	3 (100%)	6 (46.2%)	12 (63.2%)	4 (100%)	46 (58.2%)	68 (57.6%)
*Inner context*						
Unsure of the evidence^&^	0 (0%)	3 (23.1%)	3 (15.8%)	0 (0%)	2 (2.5%)	8 (6.8%)
Internal leadership opposition^&^	1 (33.3%)	4 (30.8%)	6 (31.6%)	0 (0%)	6 (7.6%)	17 (14.4%)
Limited awareness of need from staff/leadership^&^	1 (33.3%)	2 (15.4%)	5 (26.3%)	0 (0%)	9 (11.4%)	17 (14.4%)
Limited interest from SSP clients^&^	0 (0%)	1 (7.7%)	0 (0%)	0 (0%)	0 (0%)	1 (0.8%)
Unsure of best practices for implementation^&^	0 (0%)	0 (0%)	3 (15.8%)	1 (25.0%)	5 (6.3%)	9 (7.6%)
Missing	0 (0%)	0 (0%)	0 (0%)	0 (0%)	5 (6.3%)	5 (4.2%)

^a^Percentage (%) values may not add up to 100% due to rounding

^b^Includes: Illinois, Indiana, Iowa, Kansas, Michigan, Minnesota, Missouri, Nebraska, North Dakota, Ohio, South Dakota, Wisconsin

^c^Includes: Connecticut, Maine, Massachusetts, New Hampshire, New Jersey, New York, Pennsylvania, Rhode Island, Vermont

^d^Includes: Alaska, Arizona, California, Colorado, Hawaii, Idaho, Montana, Nevada, New Mexico, Oregon, Utah, Washington, Wyoming

^e^Includes: Alabama, Arkansas, Delaware, Washington D.C., Florida, Georgia, Kentucky, Louisiana, Maryland, Mississippi, North Carolina, Oklahoma, South Carolina, Tennessee, Texas, Virginia, West Virginia, Puerto Rico

^f^Defined as tribal affiliated organization, academic healthcare organization, or private or commercial health organization

^g^Defined as university funding or other

^h^This question was only asked of organizations that provide syringes (*n* = 109)

^*^P-value is for Fisher’s exact test due to low cell count (≤ 5 observations)

^$^ Variable was ‘select all that apply’ and therefore cell counts add up to greater than the total number of observations

^&^ ‘Select all that apply’ exposures (e.g., urbanicity) were analyzed as separate binary indicator variables (i.e., presence vs. absence of that category). Because respondents could select multiple categories, each *p*-value reflects a comparison between those who selected the given category and those who did not (e.g., urban vs. non-urban)

*SSP* Syringe services program, *IQR* Interquartile range, *HIV* Human immunodeficiency virus, *PrEP* HIV pre-exposure prophylaxis, *PEP* = *HIV* post-exposure prophylaxis, *STI* Sexually transmitted infection, *SSP* Syringe services program, *MOUD* Medications for opioid use disorder

**Fig. 1:**
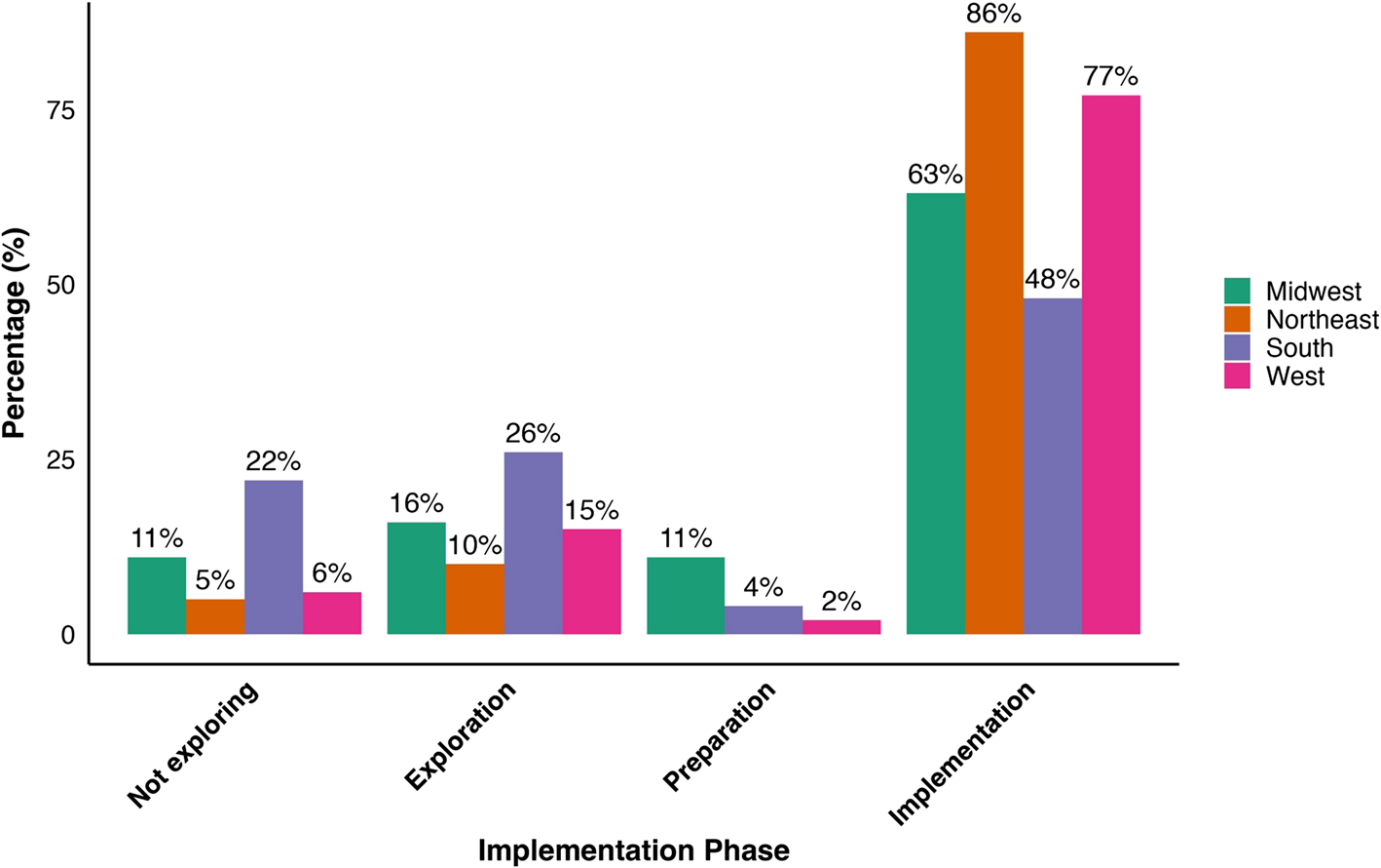
Regional differences in safer smoking supply implementation across the United States, November–December 2023 (*n* = 115). Notes: Percentage (%) values represent row percents and may not add up to 100% due to rounding; *n* = 3 organizations that did not specify their implementation phase were removed for clarity

Figure 1 outlines regional differences in safer smoking supply implementation. Most organizations in the Midwest (63%) were providing safer smoking supplies, with 11% preparing to implement in the next 6 months, 16% exploring implementation, and 11% not exploring implementation. Nearly all organizations in the Northeast (86%) were in implementation, while far fewer in this region were exploring (10%) and not exploring (5%) implementation; none were preparing to implement in the next 6 months. The South had relatively less organizations in the implementation phase (48%) compared to other regions and was relatively split between exploration (26%) and not exploring (22%) implementation. Far fewer organizations in the U.S. South were in preparation (4%). In the West, over three-fourths (77%) of organizations were in implementation, while less were in preparation (2%), exploration (15%), and not exploring implementation (6%).

### Implementation determinants of safer smoking supplies among all organizations

In unadjusted Poisson regression models (Table 2), organizations from the Northeast (PR = 1.27; 95% CI: 1.09, 1.47) and West (PR = 1.20; 95% CI: 1.03, 1.38) were more likely to have implemented safer smoking supplies compared to organizations from the U.S. South. Serving exurban/semi-rural communities (PR = 1.14; 95% CI: 1.03, 1.25) compared to not serving exurban/semi-rural communities, being a CBO (PR = 1.20; 95% CI: 1.06, 1.37) compared to not being a CBO, and receiving foundation funding (PR = 1.14; 95% CI: 1.04, 1.26) and private donations from fundraising (PR = 1.12; 95% CI: 1.01, 1.24) compared to not receiving these sources of funding were also positively associated with safer smoking supply implementation. Organizations currently offering syringes (PR = 1.40; 95% CI: 1.11, 1.75) and other injection alternatives, including safer snorting (PR = 1.43; 95% CI: 1.30, 1.58) and anal administration supplies (PR = 1.24; 95% CI: 1.12, 1.37) were also more likely to have implemented safer smoking supplies (compared to not offering these services). On the other hand, receiving federal funding (PR = 0.89; 95% CI: 0.80, 0.98) compared to not receiving federal funding, being unsure about changing community demand for safer smoking supplies (compared to perceiving that demand has not changed; PR = 0.65; 95% CI: 0.55, 0.78), opposition external to the organization (PR = 0.86; 95% CI: 0.75, 0.98), fear of external pushback (PR = 0.87; 95% CI: 0.77, 0.99) and facing internal leadership opposition (PR = 0.79; 95% CI: 0.66, 0.94) were all negatively associated with the safer smoking supply implementation (compared to not experiencing these barriers).

**Table 2:** Bivariate relationship between safer smoking supplies implementation and SSP characteristics in the United States, November–December 2023 (*n* = 118)

	**Has not implemented smoking supplies (*n* = 39; 33.1%)**	**Has implemented smoking supplies (*n* = 79; 66.9%)**	**Total (*n* = 118)**^**a**^	***P*-value**^**b**^	**Unadjusted Prevalence Ratio (95% CI)**^**c**^
**Organizational Characteristics**					
**Region**					
Midwest^d^	7 (17.9%)	12 (15.2%)	19 (16.1%)	0.006*	1.11 (0.93, 1.34)
Northeast^e^	3 (7.7%)	18 (22.8%)	21 (17.8%)		1.27 (1.09, 1.47)
West^f^	12 (30.8%)	36 (45.6%)	48 (40.7%)		1.20 (1.03, 1.38)
South^g^	15 (38.5%)	13 (16.5%)	28 (23.7%)		Ref
**Urbanicity of areas served**^**$**^					
Urban^&^	22 (56.4%)	51 (64.6%)	73 (61.9%)	0.693	–
Suburban^&^	10 (25.6%)	27 (34.2%)	37 (31.4%)	0.643	–
Exurban/Semi-rural^&^	5 (12.8%)	25 (31.6%)	30 (25.4%)	0.042*	1.14 (1.03, 1.25)
Rural^&^	13 (33.3%)	33 (41.8%)	46 (39.0%)	0.676	–
**Type of Organization**^**$**^					
Community-based Organization/Non-profit^&^	22 (56.4%)	65 (82.3%)	87 (73.7%)	0.011	1.20 (1.06, 1.37)
City, County, or State Health Department^&^	15 (38.5%)	16 (20.3%)	31 (26.3%)	0.107	–
Other^h^^,&^	3 (7.7%)	3 (3.8%)	6 (5.1%)	0.395*	–
**Funding sources**^**$**^					
Federal^&^	24 (61.5%)	31 (39.2%)	55 (46.6%)	0.074	0.89 (0.80, 0.98)
State^&^	32 (82.1%)	66 (83.5%)	98 (83.1%)	0.980	–
Foundation^&^	11 (28.2%)	42 (53.2%)	53 (44.9%)	0.037	1.14 (1.04, 1.26)
Private donations from fundraising^&^	13 (33.3%)	43 (54.4%)	56 (47.5%)	0.097	1.12 (1.01, 1.24)
Other^i^^,&^	11 (28.2%)	22 (27.8%)	33 (28.0%)	0.929	–
**Duration of operation**					
Less than one year	0 (0%)	2 (2.5%)	2 (1.7%)	0.816*	–
1–2 years	1 (2.6%)	5 (6.3%)	6 (5.1%)		
3–5 years	8 (20.5%)	16 (20.3%)	24 (20.3%)		
Greater than 5 years	30 (76.9%)	55 (69.6%)	85 (72.0%)		
**Number of unique clients (past month)**					
Median [IQR]	165 [288]	300 [551]	300 [499]	0.175	–
Unsure or missing	16 (41.0%)	20 (25.3%)	36 (30.5%)		
**Services offered**^**$**^					
Naloxone^&^	38 (97.4%)	79 (100%)	117 (99.2%)	0.360	–
Syringes^&^	32 (82.1%)	77 (97.5%)	109 (92.4%)	0.012	1.40 (1.11, 1.75)
Safer snorting supplies^&^	4 (10.3%)	60 (75.9%)	64 (54.2%)	< 0.001*	1.43 (1.30, 1.58)
Anal administration supplies^&^	10 (25.6%)	52 (65.8%)	62 (52.5%)	< 0.001	1.24 (1.12, 1.37)
Basic first aid^&^	35 (89.7%)	74 (93.7%)	109 (92.4%)	0.751	–
Food^&^	18 (46.2%)	52 (65.8%)	70 (59.3%)	0.123	–
Safer sex supplies^&^	37 (94.9%)	77 (97.5%)	114 (96.6%)	0.764	–
HIV testing^&^	28 (71.8%)	56 (70.9%)	84 (71.2%)	0.995	–
Hepatitis C testing^&^	26 (66.7%)	51 (64.6%)	77 (65.3%)	0.975	–
PrEP or PEP for HIV^&^	14 (35.9%)	27 (34.2%)	41 (34.7%)	0.983	–
HIV treatment^&^	7 (17.9%)	17 (21.5%)	24 (20.3%)	0.902	–
Hepatitis C treatment^&^	8 (20.5%1.)	19 (24.1%)	27 (22.9%)	0.912	–
STI testing^&^	17 (43.6%)	41 (51.9%)	58 (49.2%)	0.697	–
STI treatment^&^	15 (38.5%)	23 (29.1%)	38 (32.2%)	0.593	–
Basic clinical services^&^	15 (38.5%)	27 (34.2%)	42 (35.6%)	0.901	–
Mental health services^&^	15 (38.5%)	20 (25.3%)	35 (29.7%)	0.339	–
Referrals^&^	35 (89.7%)	71 (89.9%)	106 (89.8%)	1.000	–
Case management/housing coordination services^&^	18 (46.2%)	41 (51.9%)	59 (50.0%)	0.842	–
MOUD coordination^&^	23 (59.0%)	44 (55.7%)	67 (56.8%)	0.944	–
**Duration of providing syringes**^**j**^				0.951*	–
Less than one year	2 (5.1%)	3 (3.8%)	5 (4.2%)		
1–2 years	3 (7.7%)	8 (10.1%)	11 (9.3%)		
3–5 years	9 (23.1%)	18 (22.8%)	27 (22.9%)		
Greater than 5 years	18 (46.2%)	44 (55.7%)	62 (52.5%)		
Unsure or missing	7 (17.9%)	6 (7.6%)	13 (11.0%)		
**Implementation Determinants**					
**Perceived change in demand for smoking supplies (past 6 months)**				< 0.001*	
Increased	19 (48.7%)	59 (74.7%)	78 (66.1%)		1.06 (0.93, 1.21)
Decreased	0 (0.00%)	0 (0.00%)	0 (0.00%)		N/A
Unsure	12 (30.8%)	1 (1.3%)	13 (11.0%)		0.65 (0.55, 0.78)
Remained the same	8 (20.5%)	15 (19.0%)	23 (19.5%)		Ref
**Barriers to implementation**^**$**^					
*Outer context*					
Local policies^&^	21 (53.8%)	27 (34.2%)	48 (40.7%)	0.123	–
Federal policies^&^	13 (33.3%)	24 (30.4%)	37 (31.4%)	0.948	–
External opposition^&^	15 (38.5%)	14 (17.7%)	29 (24.6%)	0.048	0.86 (0.75, 0.98)
Fear of external pushback^&^	17 (43.6%)	18 (22.8%)	35 (29.7%)	0.067	0.87 (0.77, 0.99)
Insufficient funding^&^	22 (56.4%)	46 (58.2%)	68 (57.6%)	0.982	–
*Inner context*					
Unsure of the evidence^&^	6 (15.4%)	2 (2.5%)	8 (6.8%)	0.155*	–
Internal leadership opposition^&^	11 (28.2%)	6 (7.6%)	17 (14.4%)	0.011	0.79 (0.66, 0.94)
Limited awareness of need from staff/leadership^&^	8 (20.5%)	9 (11.4%)	17 (14.4%)	0.415	–
Limited interest from SSP clients^&^	1 (2.6%)	0 (0%)	1 (0.8%)	0.331*	–
Unsure of best practices for implementation^&^	4 (10.3%)	5 (6.3%)	9 (7.6%)	0.476*	–
Missing	0 (0.0%)	5 (6.3%)	5 (4.2%)	0.169*	–

^a^ Percentage (%) values may not add up to 100% due to rounding

^b^ P-values are from chi-square and Fisher’s exact tests (for variables with cell counts ≤ 5) for categorical variables and Students t-tests for continuous variables

^c^ Prevalence ratios and 95% confidence intervals were estimated using modified Poisson regression with ‘has not implemented safer smoking supplies’ as the reference group

^d^ Includes: Illinois, Indiana, Iowa, Kansas, Michigan, Minnesota, Missouri, Nebraska, North Dakota, Ohio, South Dakota, Wisconsin

^e^ Includes: Connecticut, Maine, Massachusetts, New Hampshire, New Jersey, New York, Pennsylvania, Rhode Island, Vermont

^f^ Includes: Alaska, Arizona, California, Colorado, Hawaii, Idaho, Montana, Nevada, New Mexico, Oregon, Utah, Washington, Wyoming

^g^ Includes: Alabama, Arkansas, Delaware, Washington D.C., Florida, Georgia, Kentucky, Louisiana, Maryland, Mississippi, North Carolina, Oklahoma, South Carolina, Tennessee, Texas, Virginia, West Virginia, Puerto Rico

^h^ Defined as tribal affiliated organization, academic healthcare organization, or private or commercial health organization

^i^ Defined as university funding or other

^j^ This question was only asked of organizations that provide syringes (n = 109)

^*^ P-value is for Fisher’s exact test due to low cell count (≤ 5 observations)

^$^ Variable was ‘select all that apply’ and therefore cell counts add up to greater than the total number of observations

^&^ ‘Select all that apply’ exposures (e.g., urbanicity) were analyzed as separate binary indicator variables (i.e., presence vs. absence of that category). Because respondents could select multiple categories, each p-value reflects a comparison between those who selected the given category and those who did not (e.g., urban vs. non-urban)

*SSP *Syringe services program, *IQR *Interquartile range, *HIV *Human immunodeficiency virus, *PrEP = HIV *pre-exposure prophylaxis, *PEP *HIV post-exposure prophylaxis, *STI *Sexually transmitted infection, *MOUD * Medications for opioid use disorder

### Implementation and sustainment considerations among organizations that have implemented safer smoking supplies

Most organizations that had implemented safer smoking supplies were motivated to do so by request/feedback from SSP clients (84%; Table 3). Common facilitators of safer smoking supply implementation included predominantly inner-contextual factors like making safer smoking supplies an organizational priority (65%) and internal leadership support (57%).

**Table 3:** Implementation and sustainment considerations for safer smoking supplies (*n* = 79)

	**Total (*n* = 79)**^**a**^
**Implementation Determinants**	
**Motivations for providing safer smoking supplies**^**$**^	
Request/feedback from SSP clients	66 (83.5%)
Request/suggestion from a local harm reduction partner	18 (22.8%)
Guidance/technical assistance	27 (34.2%)
Funding opportunity	20 (25.3%)
Other specific motivation^b^	19 (24.1%)
Missing	3 (3.8%)
**Facilitators of implementation**	
*Outer context*	
New funding specifically for safer smoking supplies	22 (27.8%)
New funding not specific for safer smoking supplies	27 (34.2%)
External support (e.g., from local health department)	15 (19.0%)
Collaborator/partner support	17 (21.5%)
*Inner context*	
Internal leadership support	45 (57.0%)
Organization made it a priority	51 (64.6%)
Missing	5 (6.3%)
**Additional Safer Smoking Supply Measures**	
**Duration of providing safer smoking supplies**	
Less than one year	14 (17.7%)
1–2 years	32 (40.5%)
3–5 years	19 (24.1%)
Greater than 5 years	9 (11.4%)
**Safer smoking supplies provided**	
Bubble pipes/oil burners	61 (77.2%)
Hammer pipes	36 (45.6%)
Straight pipes	63 (79.7%)
Mouthpieces	67 (84.8%)
Screens/filters	63 (79.7%)
Foil	50 (63.3%)
Push sticks	45 (57.0%)
Lighters	14 (17.7%)
Matches	5 (6.3%)
Lip balm	65 (82.3%)
Alcohol swabs	72 (91.1%)
Safer smoking classes	7 (8.9%)
Educational materials on safer smoking practices	57 (72.2%)
Missing	3 (3.8%)
**Provides any type of pipe**^**c**^	68 (86.1%)
Missing	3 (3.8%)
**Number of pipes distributed (past month)**^**d**^	
Median [IQR]	500 [910]
Missing	24 (30.4%)
**Sets limit on number of pipes per encounter**^**d**^	
No	17 (21.5%)
Yes	49 (62.0%)
**Limit on number of pipes per encounter**^**e**^	
Median [IQR]	2 ( 1)
Missing	32 (40.5%)
**Asks participants to exchange used pipes for new ones**^**d**^	
No	60 (75.9%)
Yes	7 (8.9%)
**Sustainment and Scale-up Considerations**	
**Provides sufficient quantities of safer smoking supplies to meet community needs**	
Disagree	39 (49.4%)
Agree	34 (43.0%)
**Offering safer smoking supplies are consistent with mission**	
Disagree	4 (5.1%)
Agree	69 (87.3%)
**Has a mix of stable and flexible funding for safer smoking supplies**	
Disagree	40 (50.6%)
Agree	30 (38.0%)
**Has sufficient funding for safer smoking supplies**	
Disagree	46 (58.2%)
Agree	26 (32.9%)
**Community is invested in safer smoking supply program**	
Disagree	30 (38.0%)
Agree	41 (51.9%)
**Safer smoking supplies are well-integrated into operations**	
Disagree	11 (13.9%)
Agree	61 (77.2%)
**Safer smoking supply program can be evaluated appropriately**	
Disagree	9 (11.4%)
Agree	60 (75.9%)

^a^Percentage (%) values may not add up to 100% due to rounding; Total column Ns may not sum to totals due to missing values

^b^Other specific motivations included wanting to expand harm reduction offerings, seeing a need for safer smoking supplies, staff lived experience with substance use, benefits of safer smoking supplies (e.g., reduced risk of overdose, transmission of infectious diseases), and feeling like offering safer smoking supplies “was the right thing to do.”

^c^Defined as providing bubble pipe/ “oil burner,” hammer, or straight pipes

^d^This question was only asked of organizations that provide pipes (*n* = 68)

^e^This question was only asked of organizations that set limits on the number of pipes provided per encounter (*n* = 49)

*SSP* Syringe services program, *IQR* Interquartile range

On average, organizations had implemented safer smoking supplies more recently than syringes (1–2 years vs. > 5 years ago). Regarding specific safer smoking supplies, most organizations provided alcohol swabs (91%), mouthpieces (85%), straight pipes (80%), screens/filters (80%), lip balm (82%), bubble pipes/ “oil burners” (77%), educational materials on safer smoking practices (72%), foil (63%), and push sticks (57%). Among the 68 (86%) organizations providing any type of pipe, the median number distributed in the past month was 500 (IQR: 910) and most limited the number of pipes per encounter (62%) but did not require participants to exchange used pipes for new ones (76%). For those limiting the number of pipes per encounter (n = 49), the median limit was 2.00 (IQR: 1.00).

Most respondents from organizations that had implemented safer smoking supplies believed that safer smoking supplies aligned with their organization’s mission (87%), their communities were invested in their safer smoking supply program (52%), safer smoking supply distribution was well-integrated into their operations (77%) and that they possessed the capacity to evaluate their programs appropriately (76%). However, substantial proportions of these organizations’ respondents believed that their organizations were not providing sufficient quantities of safer smoking supplies to meet community needs (49%) and lacked sufficient amounts of funding (58%) or mixes of stable and flexible funding necessary to sustain their safer smoking supply program (51%).

## Discussion

Nationally, SSPs’ efforts to reach PWUD with critical preventative services while being responsive to community needs have led to the increased adoption and implementation of safer smoking supplies [19]. The real-world implementation of safer smoking supplies has rapidly outpaced our understanding of them as an effective intervention to reduce negative health outcomes for PWUD. Our study provides the first comprehensive assessment of safer smoking supply implementation within U.S. SSPs, which, drawing from the EPIS framework [25], highlights notable inner- and outer-contextual implementation determinants that may be relevant to the adoption, implementation and sustainment of these services. Ultimately, drivers of safer smoking supply implementation were based on participant need for the service and inner-setting facilitators like SSP flexibility and leadership support; yet were noticeably constrained by outer-setting funding opportunities and federal/regional policies.

In alignment with the exploration phase of EPIS [25], most organizations in our sample recognized the growing need for safer smoking supplies, with two-thirds of survey respondents perceiving an increased demand for these supplies in the past six months (and no respondents perceiving decreased demand). However, there was a disproportionate number of city, county, or state-sponsored SSPs from the Southern U.S. that were not exploring safer smoking supply implementation. While this may reflect regional differences in underlying substance use epidemiology and routes of administration [5], it may also reflect community and outer contextual barriers to implementation, such as stigma towards smoking supplies, available funding for safer smoking supply implementation and laws prohibiting their distribution (i.e., drug paraphernalia statutes). Notably, these SSPs also disproportionately reported fear of community pushback as a barrier to safer smoking supply implementation, giving credence to this latter interpretation. This notion has also held true for implementation of other prevention interventions, like SSPs in general, which have limited their implementation in the U.S. South [32]. These findings underscore the misalignment and interaction between individual consumers (i.e. PWUD), organizational context, and outer contextual determinants that significantly influences the implementation process for safer smoking supplies.

That said, as shifts in drug consumption behaviors became apparent to SSP personnel, mainly in the past two years, our findings suggest that SSPs dynamically adapted their organizational priorities to prepare for the implementation of safer smoking supplies. Harm reduction practitioners often adopt new services to meet clients’ needs before supportive policies or funding mechanisms are available [33]. Such has been the case with many harm reduction interventions, including safer consumption sites in Canada [34] and the U.S., where they remain illegal federally despite substantial evidence of their effectiveness [35, 36]. In addition, SSPs that had the capacity to implement other injection alternatives, such as safer snorting and anal administration supplies, appeared to be more prepared to implement safer smoking supplies. This ability to pivot and expand services reflects these organizations' inherent responsiveness and flexibility in meeting the evolving needs of PWUD. Yet, until this assessment, it remained unclear what characteristics were associated with the flexibility necessary to facilitate the implementation of safer smoking supplies.

We found that SSPs classified as CBOs (vs. health departments) were more likely to have implemented safer smoking supplies, possibly due to CBOs’ greater freedom from policy restrictions that may prohibit health department-based SSPs from purchasing or distributing these supplies. This aligns with recent research that showed safer smoking supply implementation at SSPs was associated with identifying as a CBO (as compared to a health department) [19]. CBOs’ implementation of more comprehensive harm reduction services also transcends safer smoking supplies. CBO SSPs with government funding have been shown to distribute more syringes and naloxone than health department-based SSPs; and CBO SSPs are more likely to implement fentanyl test strips and buprenorphine regardless of their source of funding (e.g., governmental or non-governmental) [37], which are other relatively new services for SSPs. As such, SSPs classified as CBOs may be able to more readily implement innovative interventions like safer smoking supplies and other injection alternatives [19], as was suggested during the COVID-19 pandemic when traditional in-person services were severely disrupted [33]. However, despite evidence that a majority of SSPs in our sample were able to adapt and implement safer smoking supplies in the context of shifting community needs, we also noted several multi-level challenges that organizations faced with implementation.

We identified several specific outer-contextual constraints on safer smoking supply implementation. First, we found that SSPs from the Northeastern and Western U.S. were more likely to have implemented safer smoking supplies, which may reflect regional differences in safer smoking supply need [5], state-level paraphernalia laws, harm reduction policies, and political climates as noted above [38, 39]. Interestingly, we also found that SSPs based in exurban/semi-rural communities were more likely to distribute safer smoking supplies. While this somewhat contradicts recent findings that increasing SSP budgets and service opportunities were associated with increasing urbanization [40] and that an SSPs’ total annual budget was not related to safer smoking supply distribution [19], this could indicate that flexible funding, not simply more funding, is a necessary precursor to safer smoking supply implementation. Additional qualitative research with SSPs from diverse communities will be critical to understanding more of these nuanced outer-contextual implementation determinants, especially in the context of sparse data for certain implementation phases outlined here.

Second, we found that SSPs receiving federal funding were less than half as likely to have implemented safer smoking supplies. Although federal funding for SSPs has expanded recently [41], federal funds cannot be used to purchase sterile glass pipes, which are critical components of safer smoking supply kits. Glass pipes can be costly and potentially result in limits on the number of pipes distributed per encounter, such as we saw in this study. As highlighted by SSP personnel in our sample, current coverage levels of glass pipes are likely insufficient to meet community needs and prevent health harms from equipment sharing (e.g., hepatitis C) and inhalation of make-shift supplies like plastic bottles and cans, suggesting that SSPs could benefit from the removal of restrictions on federal funding for purchasing safer smoking supplies. This further supports our interpretation that flexible and diverse funding sources may be a more significant determinant than additional funding in facilitating safer smoking supply implementation at SSPs in the U.S.

While most SSPs in our sample have successfully implemented safer smoking supplies, substantial challenges remain in ensuring their reach, sustainability and scale-up. Although most respondents felt that safer smoking supplies aligned with their organization's mission and had strong community support, nearly half indicated they were not providing sufficient quantities to meet demand, and over half expressed concerns about inadequate and unstable funding. Together with our other findings, these insights highlight the need for more robust and flexible funding mechanisms to sustain and scale safer smoking supplies, especially at non-CBO and predominantly federally funded organizations. As SSPs continue to navigate the complexities of evolving drug use-related challenges for PWUD, addressing these funding and supply challenges will be crucial to maintaining the momentum in safer smoking supply distribution and optimizing its public health impact.

Several limitations of this study should be noted. First, due to our reliance on self-report, our survey respondents may have over- or underreported specific practices or challenges. Furthermore, because respondents could have held any (or many) roles within their organization, some may have been less familiar with safer smoking supply implementation efforts, providing less accurate information and opinions. Second, because our study was cross-sectional, we cannot draw causal inferences about the actual impacts of specific factors on safer smoking supply implementation. Third, our sample was relatively small, which led to sparse data precluding more advanced analyses by implementation phase. Importantly, it is unlikely that our sample is representative all U.S. SSPs, and we lack information on programs that did not receive or respond to our survey. As there is no detailed publicly-available dataset describing the universe of U.S. SSPs, we are unable to estimate bias from non-response or apply survey weights to account for potential imbalances. According to available estimates, we estimate that we may have sampled approximately 20% of known U.S. SSPs [42]. Larger, more representative studies are needed. Finally, while EPIS provided a useful structure for surveying SSPs about their safer smoking supply implementation efforts, due to feasibility considerations, our survey was limited in length and we may have missed relevant implementation factors or the complex interactions between them. Future research, including larger longitudinal studies and in-depth qualitative investigations, are needed to expand on the preliminary findings provided here and more thoroughly investigate sustainability considerations.

## Conclusions

We found that the majority of U.S. SSPs responding to our survey had implemented safer smoking supplies as an emerging intervention to address the rising prevalence of smoking unregulated drugs. However, significant barriers to implementation exist, particularly outer contextual factors (e.g., local opposition, funding restrictions) that constrain organizations' operations. Despite these obstacles, however, national data on drug consumption behaviors underscore the need to support SSPs’ implementation and optimization of safer smoking supply distribution through stable and flexible funding mechanisms, supportive policies, and community engagement. Addressing the intersection of public health crises resulting from unregulated drug use will require additional implementation research on effective implementation strategies and programmatic supports for this critical intervention within this delivery setting.

## Data Availability

The dataset used and/or analyzed during the current study is available from the corresponding author, Angela R. Bazzi (abazzi@health.ucsd.edu), upon reasonable request.

## Abbreviations

SSP: Syringe Services Program
CBO: Community-Based Organization
EPIS: Exploration, Preparation, Implementation, Sustainment (Framework)
PWUD: People Who Use Drugs
HIV: Human Immunodeficiency Virus
HCV: Hepatitis C Virus
STI: Sexually Transmitted Infection
PrEP: HIV Pre-Exposure Prophylaxis
PEP: HIV Post-Exposure Prophylaxis

## References

[1] Ciccarone D. The rise of illicit fentanyls, stimulants and the fourth wave of the opioid overdose crisis. Curr Opin Psychiatry. 2021;34(4):344–50. 33965972 10.1097/YCO.0000000000000717 PMC8154745

[2] Friedman J, Shover CL. Charting the fourth wave: Geographic, temporal, race/ethnicity and demographic trends in polysubstance fentanyl overdose deaths in the United States, 2010–2021. Addiction. 2023;118(12):2477–85. 37705148 10.1111/add.16318

[3] Gonsalves GS, Paltiel AD, Thornhill T, DeMaria A Jr, Cranston K, Klevens RM, et al. Patterns of Infectious Disease Associated With Injection Drug Use in Massachusetts. Clin Infect Dis. 2023;76(12):2134–9. 36757712 10.1093/cid/ciad073 PMC10273381

[4] Friedman JR, Abramovitz D, Skaathun B, Rangel G, Harvey-Vera A, Vera CF, et al. Illicit Fentanyl Use and Hepatitis C Virus Seroconversion Among People Who Inject Drugs in Tijuana and San Diego: Results From a Binational Cohort Study. Clinical Infectious Diseases. 2024. 10.1093/cid/ciae372 PMC11478577 39078273

[5] Karandinos G, Unick J, Ondocsin J, Holm N, Mars S, Montero F, et al. Decrease in injection and rise in smoking and snorting of heroin and synthetic opioids, 2000–2021. Drug and Alcohol Dependence. 2024:111419. 10.1016/j.drugalcdep.2024.111419 PMC11684856 39216201

[6] Tanz L, Gladden RM, Dinwiddie AT, et al. Routes of Drug Use Among Drug Overdose Deaths – United States, 2020–2022. MMWR Morb Mortal Wkly Rep. 2024;73:124–30. 38358969 10.15585/mmwr.mm7306a2 PMC10899081

[7] Eger WH, Abramovitz D, Bazzi AR, Bórquez A, Vera CF, Harvey-Vera A, et al. Changes in injecting versus smoking heroin, fentanyl, and methamphetamine among people who inject drugs in San Diego, California, 2020–2023. Drug Alcohol Depend. 2024;259:111318. 38692135 10.1016/j.drugalcdep.2024.111318 PMC11463215

[8] Kral AH, Lambdin BH, Browne EN, Wenger LD, Bluthenthal RN, Zibbell JE, et al. Transition from injecting opioids to smoking fentanyl in San Francisco. California Drug Alcohol Depend. 2021;227:109003. 34482046 10.1016/j.drugalcdep.2021.109003 PMC10790652

[9] Parent S, Papamihali K, Graham B, Buxton JA. Examining prevalence and correlates of smoking opioids in British Columbia: opioids are more often smoked than injected. Substance Abuse Treatment, Prevention, and Policy. 2021;16(1):79. 34663374 10.1186/s13011-021-00414-6 PMC8522853

[10] Megerian CE, Bair L, Smith J, Browne EN, Wenger LD, Guzman L, et al. Health risks associated with smoking versus injecting fentanyl among people who use drugs in California. Drug Alcohol Depend. 2024;255:111053. 38128362 10.1016/j.drugalcdep.2023.111053

[11] Ciccarone D, Holm N, Ondocsin J, Schlosser A, Fessel J, Cowan A, et al. Innovation and adaptation: The rise of a fentanyl smoking culture in San Francisco. PLoS One. 2024;19(5):e0303403. 38776268 10.1371/journal.pone.0303403 PMC11111043

[12] Mackey KM, Beech EH, Williams BE, Anderson JK, Young S, Parr NJ. VA Evidence-based Synthesis Program Reports. Effectiveness of Syringe Services Programs: A Systematic Review. Washington (DC): Department of Veterans Affairs (US); 2023. 38166137

[13] Whiteman A, Burnett J, Handanagic S, Wejnert C, Broz D. Distance matters: The association of proximity to syringe services programs with sharing of syringes and injecting equipment- 17 U.S. cities, 2015. Int J Drug Policy. 2020;85:102923. 32920424 10.1016/j.drugpo.2020.102923

[14] Centers for Disease Control and Prevention. Safety and Effectiveness of Syringe Services Programs. 2024.

[15] Broz D, Carnes N, Chapin-Bardales J, Des Jarlais DC, Handanagic S, Jones CM, et al. Syringe services programs’ role in ending the HIV epidemic in the U.S.: Why We Cannot Do It Without Them. Am J Prev Med. 2021;61(5 Suppl 1):S118-s29. 34686281 10.1016/j.amepre.2021.05.044 PMC11334402

[16] Des Jarlais DC. Harm reduction in the USA: the research perspective and an archive to David Purchase. Harm Reduct J. 2017;14(1):51. 28747189 10.1186/s12954-017-0178-6 PMC5530540

[17] Singh SB-G, C; Kingston, S. Distribution of Safer Drug Smoking Supplies as a Public Health Strategy. 2022 January 2022.

[18] Fitzpatrick T, McMahan VM, Frank ND, Glick SN, Violette LR, Davis S, et al. Heroin pipe distribution to reduce high-risk drug consumption behaviors among people who use heroin: a pilot quasi-experimental study. Harm Reduct J. 2022;19(1):103. 36138407 10.1186/s12954-022-00685-7 PMC9493152

[19] Chung EO, Patel SV, Wenger LD, Humphrey JL, Sukasih A, Bluthenthal RN, et al. Association of safer smoking supply distribution with participant encounters and naloxone distribution from syringe services programs: Findings from the National Survey of Syringe Services Programs in the United States. Drug and Alcohol Dependence Reports. 2024:100317. 10.1016/j.dadr.2024.100317 PMC11773076 39877805

[20] Lambdin BH, Bluthenthal RN, Garner BR, Wenger LD, Browne EN, Morris T, et al. Organize and mobilize for implementation effectiveness to improve overdose education and naloxone distribution from syringe services programs: a randomized controlled trial. Implement Sci. 2024;19(1):22. 38419058 10.1186/s13012-024-01354-y PMC10900734

[21] Akiba CF, Patel SV, Wenger LD, Morgan-Lopez A, Zarkin GA, Orme S, et al. Systems analysis and improvement approach to improve naloxone distribution within syringe service programs: study protocol of a randomized controlled trial. Implement Sci. 2023;18(1):33. 37537665 10.1186/s13012-023-01288-x PMC10398915

[22] Lambdin BH, Wenger L, Bluthenthal R, Bartholomew TS, Tookes HE, LaKosky P, et al. How do contextual factors influence naloxone distribution from syringe service programs in the USA: a cross-sectional study. Harm Reduct J. 2023;20(1):26. 36855181 10.1186/s12954-023-00755-4 PMC9972698

[23] Bartholomew TS, Plesons M, Serota DP, Alonso E, Metsch LR, Feaster DJ, et al. Project CHARIOT: study protocol for a hybrid type 1 effectiveness-implementation study of comprehensive tele-harm reduction for engagement of people who inject drugs in HIV prevention services. Addict Sci Clin Pract. 2024;19(1):21. 38528570 10.1186/s13722-024-00447-9 PMC10964520

[24] Tookes HE, Bartholomew TS, Suarez E, Ekowo E, Ginoza M, Forrest DW, et al. Acceptability, feasibility, and pilot results of the tele-harm reduction intervention for rapid initiation of antiretrovirals among people who inject drugs. Drug Alcohol Depend. 2021;229(Pt A):109124. 34781096 10.1016/j.drugalcdep.2021.109124 PMC9102418

[25] Aarons GA, Hurlburt M, Horwitz SM. Advancing a conceptual model of evidence-based practice implementation in public service sectors. Adm Policy Ment Health. 2011;38(1):4–23. 21197565 10.1007/s10488-010-0327-7 PMC3025110

[26] Moullin JC, Dickson KS, Stadnick NA, Rabin B, Aarons GA. Systematic review of the Exploration, Preparation, Implementation, Sustainment (EPIS) framework. Implement Sci. 2019;14(1):1. 30611302 10.1186/s13012-018-0842-6 PMC6321673

[27] Luke DA, Calhoun A, Robichaux CB, Elliott MB, Moreland-Russell S. The program sustainability assessment tool: a new instrument for public health programs. Prev Chronic Dis. 2014;11:130184. 24456645 10.5888/pcd11.130184 PMC3900326

[28] Mancini JA, Marek LI. Sustaining community-based programs for families: conceptualization and measurement. Fam Relat. 2004;53(4):339–47.

[29] Aarons GA, Sklar M, Mustanski B, Benbow N, Brown CH. “Scaling-out” evidence-based interventions to new populations or new health care delivery systems. Implement Sci. 2017;12(1):111. 28877746 10.1186/s13012-017-0640-6 PMC5588712

[30] Zou G. A modified poisson regression approach to prospective studies with binary data. Am J Epidemiol. 2004;159(7):702–6. 15033648 10.1093/aje/kwh090

[31] Team RC. R: A language and environment for statistical computing. 2020. R Foundation for Statistical Computing: Vienna, Austria. 2020.

[32] Fernández-Viña MH, Prood NE, Herpolsheimer A, Waimberg J, Burris S. State Laws Governing Syringe Services Programs and Participant Syringe Possession, 2014–2019. Public Health Reports®. 2020;135(1_suppl):128S-37S. 10.1177/0033354920921817 PMC7407055 32735195

[33] Wenger LD, Kral AH, Bluthenthal RN, Morris T, Ongais L, Lambdin BH. Ingenuity and resiliency of syringe service programs on the front lines of the opioid overdose and COVID-19 crises. Transl Res. 2021;234:159–73. 33746108 10.1016/j.trsl.2021.03.011 PMC8217165

[34] Kerr T, Mitra S, Kennedy MC, McNeil R. Supervised injection facilities in Canada: past, present, and future. Harm Reduct J. 2017;14(1):28. 28521829 10.1186/s12954-017-0154-1 PMC5437687

[35] Kral AH, Davidson PJ. Addressing the Nation’s opioid epidemic: lessons from an unsanctioned supervised Injection Site in the U.S. Am J Prev Med. 2017;53(6):919–22. 28801014 10.1016/j.amepre.2017.06.010

[36] Levengood TW, Yoon GH, Davoust MJ, Ogden SN, Marshall BDL, Cahill SR, et al. Supervised injection facilities as harm reduction: A systematic review. Am J Prev Med. 2021;61(5):738–49. 34218964 10.1016/j.amepre.2021.04.017 PMC8541900

[37] Ray BR, Humphrey JL, Patel SV, Akiba CF, Bluthenthal RN, Tookes H, et al. Comparing harm reduction and overdose response services between community-based and public health department syringe service programmes using a national cross-sectional survey. The Lancet Regional Health – Americas. 2024;34. 10.1016/j.lana.2024.100757 PMC11091529 38745887

[38] Singer JA HS. Drug Paraphernalia Laws Undermine Harm Reduction. 2022 06/07/2022.

[39] Davis CS, Carr DH, Samuels EA. Paraphernalia laws, criminalizing possession and distribution of items used to consume illicit drugs, and injection-related harm. Am J Public Health. 2019;109(11):1564–7. 31536408 10.2105/AJPH.2019.305268 PMC6775926

[40] Facente SN, Humphrey JL, Akiba C, Patel SV, Wenger LD, Tookes H, et al. Funding and delivery of syringe services programs in the United States, 2022. Am J Public Health. 2024;114(4):435–43. 38478864 10.2105/AJPH.2024.307583 PMC10937606

[41] Biden-Harris Administration Announces $28 Million in Funding Opportunities for Grants Expanding Treatment Services for Substance Use Disorder [press release]. 02/02/2024 2024.

[42] North America Syringe Exchange Network (NASEN). Harm Reduction Locations 2024 [Available from: https://nasen.org/.

